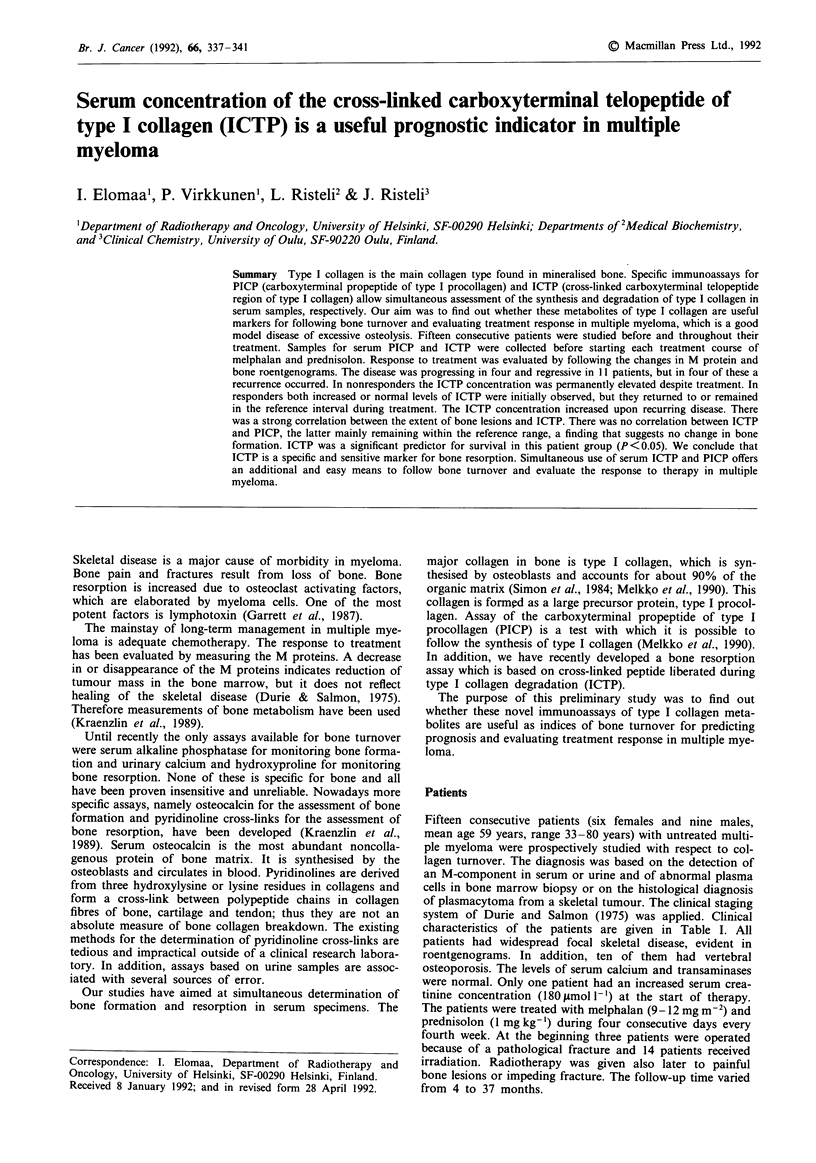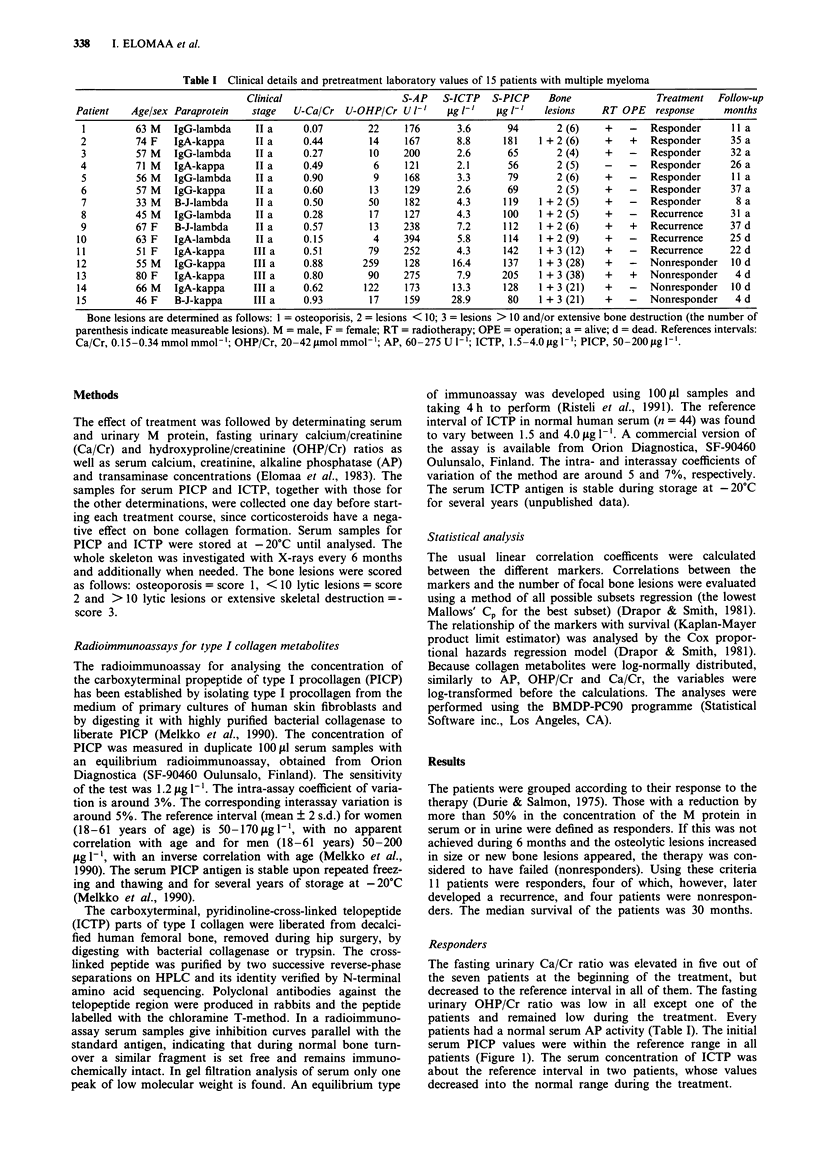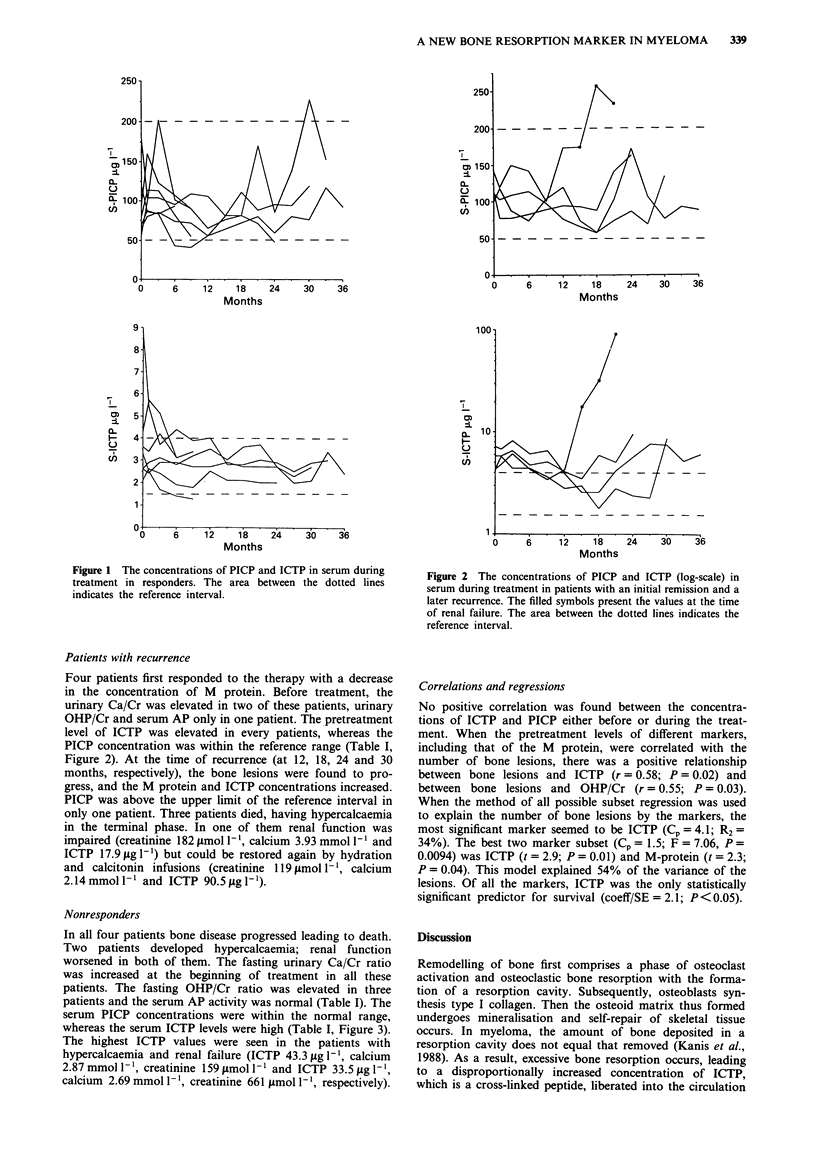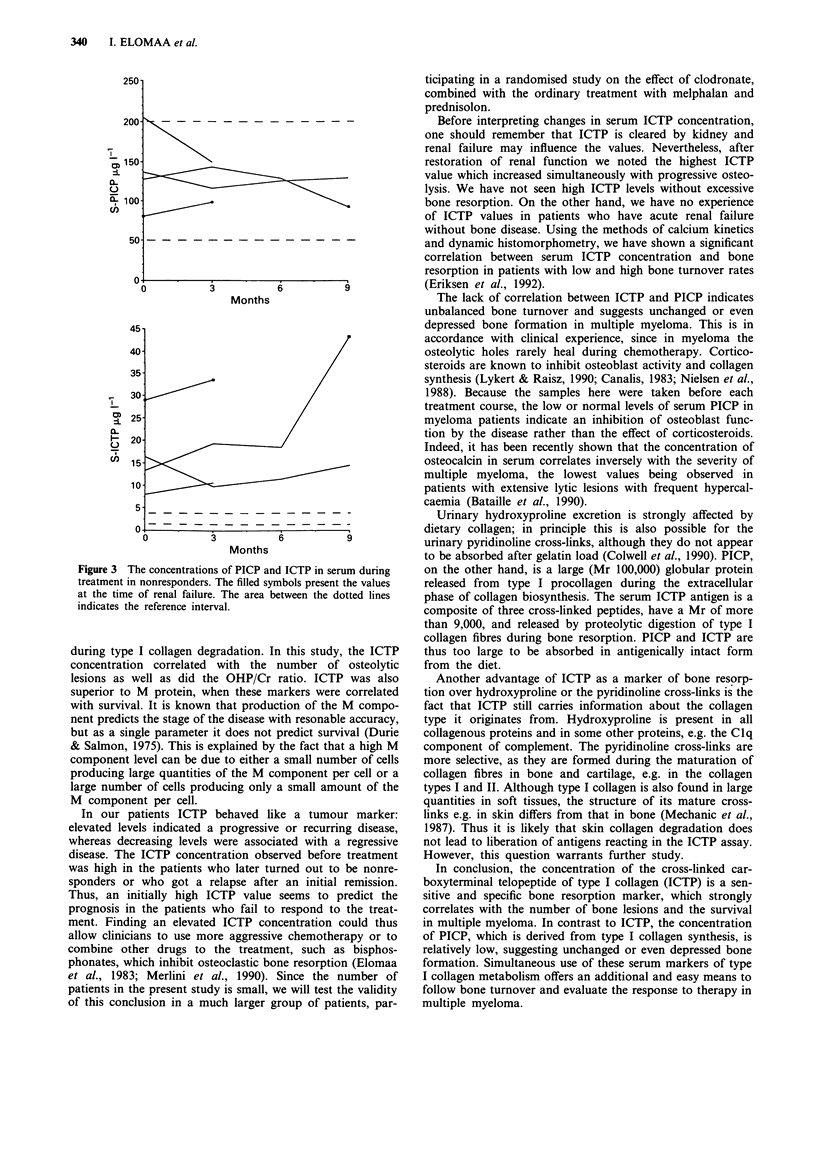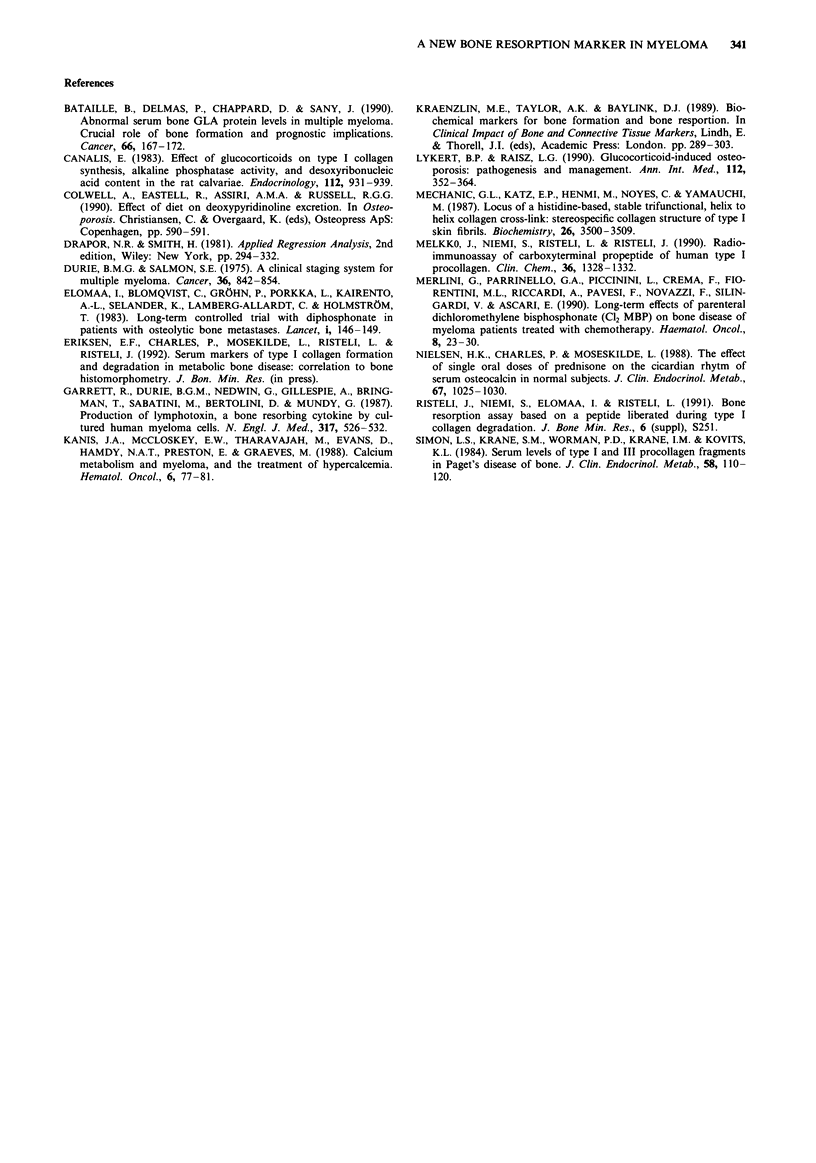# Serum concentration of the cross-linked carboxyterminal telopeptide of type I collagen (ICTP) is a useful prognostic indicator in multiple myeloma.

**DOI:** 10.1038/bjc.1992.266

**Published:** 1992-08

**Authors:** I. Elomaa, P. Virkkunen, L. Risteli, J. Risteli

**Affiliations:** Department of Radiotherapy and Oncology, University of Helsinki, Finland.

## Abstract

Type I collagen is the main collagen type found in mineralised bone. Specific immunoassays for PICP (carboxyterminal propeptide of type I procollagen) and ICTP (cross-linked carboxyterminal telopeptide region of type I collagen) allow simultaneous assessment of the synthesis and degradation of type I collagen in serum samples, respectively. Our aim was to find out whether these metabolites of type I collagen are useful markers for following bone turnover and evaluating treatment response in multiple myeloma, which is a good model disease of excessive osteolysis. Fifteen consecutive patients were studied before and throughout their treatment. Samples for serum PICP and ICTP were collected before starting each treatment course of melphalan and prednisolon. Response to treatment was evaluated by following the changes in M protein and bone roentgenograms. The disease was progressing in four and regressive in 11 patients, but in four of these a recurrence occurred. In nonresponders the ICTP concentration was permanently elevated despite treatment. In responders both increased or normal levels of ICTP were initially observed, but they returned to or remained in the reference interval during treatment. The ICTP concentration increased upon recurring disease. There was a strong correlation between the extent of bone lesions and ICTP. There was no correlation between ICTP and PICP, the latter mainly remaining within the reference range, a finding that suggests no change in bone formation. ICTP was a significant predictor for survival in this patient group (P less than 0.05). We conclude that ICTP is a specific and sensitive marker for bone resorption. Simultaneous use of serum ICTP and PICP offers an additional and easy means to follow bone turnover and evaluate the response to therapy in multiple myeloma.


Br. J. Cancer (1992), 66, 337–341 © Macmillan Press Ltd., 1992

### Serum concentration of the cross-linked carboxyterminal telopeptide of type I collagen (ICTP) is a useful prognostic indicator in multiple myeloma

I. Elomaa[1], P. Virkkunen[1], L. Risteli[2] & J. Risteli[3]

[1]*Department of Radiotherapy and Oncology, University of Helsinki, SF-00290 Helsinki; Departments of* [2]*Medical Biochemistry, and* [3]*Clinical Chemistry, University of Oulu, SF-90220 Oulu, Finland.*

**Summary** Type I collagen is the main collagen type found in mineralised bone. Specific immunoassays for PICP (carboxyterminal propeptide of type I procollagen) and ICTP (cross-linked carboxyterminal telopeptide region of type I collagen) allow simultaneous assessment of the synthesis and degradation of type I collagen in serum samples, respectively. Our aim was to find out whether these metabolites of type I collagen are useful markers for following bone turnover and evaluating treatment response in multiple myeloma, which is a good model disease of excessive osteolysis. Fifteen consecutive patients were studied before and throughout their treatment. Samples for serum PICP and ICTP were collected before starting each treatment course of melphalan and prednisolon. Response to treatment was evaluated by following the changes in M protein and bone roentgenograms. The disease was progressing in four and regressive in 11 patients, but in four of these a recurrence occurred. In nonresponders the ICTP concentration was permanently elevated despite treatment. In responders both increased or normal levels of ICTP were initially observed, but they returned to or remained in the reference interval during treatment. The ICTP concentration increased upon recurring disease. There was a strong correlation between the extent of bone lesions and ICTP. There was no correlation between ICTP and PICP, the latter mainly remaining within the reference range, a finding that suggests no change in bone formation. ICTP was a significant predictor for survival in this patient group ($P < 0.05$). We conclude that ICTP is a specific and sensitive marker for bone resorption. Simultaneous use of serum ICTP and PICP offers an additional and easy means to follow bone turnover and evaluate the response to therapy in multiple myeloma.

Skeletal disease is a major cause of morbidity in myeloma. Bone pain and fractures result from loss of bone. Bone resorption is increased due to osteoclast activating factors, which are elaborated by myeloma cells. One of the most potent factors is lymphotoxin (Garrett *et al.*, 1987).

The mainstay of long-term management in multiple myeloma is adequate chemotherapy. The response to treatment has been evaluated by measuring the M proteins. A decrease in or disappearance of the M proteins indicates reduction of tumour mass in the bone marrow, but it does not reflect healing of the skeletal disease (Durie & Salmon, 1975). Therefore measurements of bone metabolism have been used (Kraenzlin *et al.*, 1989).

Until recently the only assays available for bone turnover were serum alkaline phosphatase for monitoring bone formation and urinary calcium and hydroxyproline for monitoring bone resorption. None of these is specific for bone and all have been proven insensitive and unreliable. Nowadays more specific assays, namely osteocalcin for the assessment of bone formation and pyridinoline cross-links for the assessment of bone resorption, have been developed (Kraenzlin *et al.*, 1989). Serum osteocalcin is the most abundant noncollagenous protein of bone matrix. It is synthesised by the osteoblasts and circulates in blood. Pyridinolines are derived from three hydroxylysine or lysine residues in collagens and form a cross-link between polypeptide chains in collagen fibres of bone, cartilage and tendon; thus they are not an absolute measure of bone collagen breakdown. The existing methods for the determination of pyridinoline cross-links are tedious and impractical outside of a clinical research laboratory. In addition, assays based on urine samples are associated with several sources of error.

Our studies have aimed at simultaneous determination of bone formation and resorption in serum specimens. The major collagen in bone is type I collagen, which is synthesised by osteoblasts and accounts for about 90% of the organic matrix (Simon *et al.*, 1984; Melkko *et al.*, 1990). This collagen is formed as a large precursor protein, type I procollagen. Assay of the carboxyterminal propeptide of type I procollagen (PICP) is a test with which it is possible to follow the synthesis of type I collagen (Melkko *et al.*, 1990). In addition, we have recently developed a bone resorption assay which is based on cross-linked peptide liberated during type I collagen degradation (ICTP).

The purpose of this preliminary study was to find out whether these novel immunoassays of type I collagen metabolites are useful as indices of bone turnover for predicting prognosis and evaluating treatment response in multiple myeloma.

### Patients

Fifteen consecutive patients (six females and nine males, mean age 59 years, range 33–80 years) with untreated multiple myeloma were prospectively studied with respect to collagen turnover. The diagnosis was based on the detection of an M-component in serum or urine and of abnormal plasma cells in bone marrow biopsy or on the histological diagnosis of plasmacytoma from a skeletal tumour. The clinical staging system of Durie and Salmon (1975) was applied. Clinical characteristics of the patients are given in Table I. All patients had widespread focal skeletal disease, evident in roentgenograms. In addition, ten of them had vertebral osteoporosis. The levels of serum calcium and transaminases were normal. Only one patient had an increased serum creatinine concentration ($180\,\mu mol\,l^{-1}$) at the start of therapy. The patients were treated with melphalan ($9-12\,mg\,m^{-2}$) and prednisolon ($1\,mg\,kg^{-1}$) during four consecutive days every fourth week. At the beginning three patients were operated because of a pathological fracture and 14 patients received irradiation. Radiotherapy was given also later to painful bone lesions or impeding fracture. The follow-up time varied from 4 to 37 months.

Correspondence: I. Elomaa, Department of Radiotherapy and Oncology, University of Helsinki, SF-00290 Helsinki, Finland.
Received 8 January 1992; and in revised form 28 April 1992.

**Table I** Clinical details and pretreatment laboratory values of 15 patients with multiple myeloma

| Patient | Age/sex | Paraprotein | Clinical stage | U-Ca/Cr | U-OHP/Cr | S-AP U $l^{-1}$ | S-ICTP µg $l^{-1}$ | S-PICP µg $l^{-1}$ | Bone lesions | RT | OPE | Treatment response | Follow-up months |
|---|---|---|---|---|---|---|---|---|---|---|---|---|---|
| 1 | 63 M | IgG-lambda | II a | 0.07 | 22 | 176 | 3.6 | 94 | 2 (6) | + | – | Responder | 11 a |
| 2 | 74 F | IgA-kappa | II a | 0.44 | 14 | 167 | 8.8 | 181 | 1 + 2 (6) | + | + | Responder | 35 a |
| 3 | 57 M | IgG-lambda | II a | 0.27 | 10 | 200 | 2.6 | 65 | 2 (4) | + | – | Responder | 32 a |
| 4 | 71 M | IgA-kappa | II a | 0.49 | 6 | 121 | 2.1 | 56 | 2 (5) | – | – | Responder | 26 a |
| 5 | 56 M | IgG-lambda | II a | 0.90 | 9 | 168 | 3.3 | 79 | 2 (6) | + | – | Responder | 11 a |
| 6 | 57 M | IgG-kappa | II a | 0.60 | 13 | 129 | 2.6 | 69 | 2 (5) | + | – | Responder | 37 a |
| 7 | 33 M | B-J-lambda | II a | 0.50 | 50 | 182 | 4.3 | 119 | 1 + 2 (5) | + | – | Responder | 8 a |
| 8 | 45 M | IgG-lambda | II a | 0.28 | 17 | 127 | 4.3 | 100 | 1 + 2 (5) | + | – | Recurrence | 31 a |
| 9 | 67 F | B-J-lambda | II a | 0.57 | 13 | 238 | 7.2 | 112 | 1 + 2 (6) | + | + | Recurrence | 37 d |
| 10 | 63 F | IgA-lambda | II a | 0.15 | 4 | 394 | 5.8 | 114 | 1 + 2 (9) | + | – | Recurrence | 25 d |
| 11 | 51 F | IgA-kappa | III a | 0.51 | 79 | 252 | 4.3 | 142 | 1 + 3 (12) | + | – | Recurrence | 22 d |
| 12 | 55 M | IgG-kappa | III a | 0.88 | 259 | 128 | 16.4 | 137 | 1 + 3 (28) | + | – | Nonresponder | 10 d |
| 13 | 80 F | IgA-kappa | III a | 0.80 | 90 | 275 | 7.9 | 205 | 1 + 3 (38) | + | + | Nonresponder | 4 d |
| 14 | 66 M | IgA-kappa | III a | 0.62 | 122 | 173 | 13.3 | 128 | 1 + 3 (21) | + | – | Nonresponder | 10 d |
| 15 | 46 F | B-J-kappa | III a | 0.93 | 17 | 159 | 28.9 | 80 | 1 + 3 (21) | + | – | Nonresponder | 4 d |

Bone lesions are determined as follows: 1 = osteoporisis, 2 = lesions $<10$; 3 = lesions $>10$ and/or extensive bone destruction (the number of parenthesis indicate measureable lesions). M = male, F = female; RT = radiotherapy; OPE = operation; a = alive; d = dead. References intervals: Ca/Cr, 0.15–0.34 mmol $mmol^{-1}$; OHP/Cr, 20–42 µmol $mmol^{-1}$; AP, 60–275 U $l^{-1}$; ICTP, 1.5–4.0 µg $l^{-1}$; PICP, 50–200 µg $l^{-1}$.

### Methods

The effect of treatment was followed by determinating serum and urinary M protein, fasting urinary calcium/creatinine (Ca/Cr) and hydroxyproline/creatinine (OHP/Cr) ratios as well as serum calcium, creatinine, alkaline phosphatase (AP) and transaminase concentrations (Elomaa *et al.*, 1983). The samples for serum PICP and ICTP, together with those for the other determinations, were collected one day before starting each treatment course, since corticosteroids have a negative effect on bone collagen formation. Serum samples for PICP and ICTP were stored at −20°C until analysed. The whole skeleton was investigated with X-rays every 6 months and additionally when needed. The bone lesions were scored as follows: osteoporosis = score 1, $<10$ lytic lesions = score 2 and $>10$ lytic lesions or extensive skeletal destruction =-score 3.

### *Radioimmunoassays for type I collagen metabolites*

The radioimmunoassay for analysing the concentration of the carboxyterminal propeptide of type I procollagen (PICP) has been established by isolating type I procollagen from the medium of primary cultures of human skin fibroblasts and by digesting it with highly purified bacterial collagenase to liberate PICP (Melkko *et al.*, 1990). The concentration of PICP was measured in duplicate 100 µl serum samples with an equilibrium radioimmunoassay, obtained from Orion Diagnostica (SF-90460 Oulunsalo, Finland). The sensitivity of the test was 1.2 µg $l^{-1}$. The intra-assay coefficient of variation is around 3%. The corresponding interassay variation is around 5%. The reference interval (mean ± 2 s.d.) for women (18–61 years of age) is 50–170 µg $l^{-1}$, with no apparent correlation with age and for men (18–61 years) 50–200 µg $l^{-1}$, with an inverse correlation with age (Melkko *et al.*, 1990). The serum PICP antigen is stable upon repeated freezing and thawing and for several years of storage at −20°C (Melkko *et al.*, 1990).

The carboxyterminal, pyridinoline-cross-linked telopeptide (ICTP) parts of type I collagen were liberated from decalcified human femoral bone, removed during hip surgery, by digesting with bacterial collagenase or trypsin. The cross-linked peptide was purified by two successive reverse-phase separations on HPLC and its identity verified by N-terminal amino acid sequencing. Polyclonal antibodies against the telopeptide region were produced in rabbits and the peptide labelled with the chloramine T-method. In a radioimmunoassay serum samples give inhibition curves parallel with the standard antigen, indicating that during normal bone turnover a similar fragment is set free and remains immunochemically intact. In gel filtration analysis of serum only one peak of low molecular weight is found. An equilibrium type of immunoassay was developed using 100 µl samples and taking 4 h to perform (Risteli *et al.*, 1991). The reference interval of ICTP in normal human serum ($n = 44$) was found to vary between 1.5 and 4.0 µg $l^{-1}$. A commercial version of the assay is available from Orion Diagnostica, SF-90460 Oulunsalo, Finland. The intra- and interassay coefficients of variation of the method are around 5 and 7%, respectively. The serum ICTP antigen is stable during storage at −20°C for several years (unpublished data).

### *Statistical analysis*

The usual linear correlation coefficents were calculated between the different markers. Correlations between the markers and the number of focal bone lesions were evaluated using a method of all possible subsets regression (the lowest Mallows' $C_p$ for the best subset) (Drapor & Smith, 1981). The relationship of the markers with survival (Kaplan-Mayer product limit estimator) was analysed by the Cox proportional hazards regression model (Drapor & Smith, 1981). Because collagen metabolites were log-normally distributed, similarly to AP, OHP/Cr and Ca/Cr, the variables were log-transformed before the calculations. The analyses were performed using the BMDP-PC90 programme (Statistical Software inc., Los Angeles, CA).

### Results

The patients were grouped according to their response to the therapy (Durie & Salmon, 1975). Those with a reduction by more than 50% in the concentration of the M protein in serum or in urine were defined as responders. If this was not achieved during 6 months and the osteolytic lesions increased in size or new bone lesions appeared, the therapy was considered to have failed (nonresponders). Using these criteria 11 patients were responders, four of which, however, later developed a recurrence, and four patients were nonresponders. The median survival of the patients was 30 months.

### *Responders*

The fasting urinary Ca/Cr ratio was elevated in five out of the seven patients at the beginning of the treatment, but decreased to the reference interval in all of them. The fasting urinary OHP/Cr ratio was low in all except one of the patients and remained low during the treatment. Every patients had a normal serum AP activity (Table I). The initial serum PICP values were within the reference range in all patients (Figure 1). The serum concentration of ICTP was about the reference interval in two patients, whose values decreased into the normal range during the treatment.

**Figure 1** The concentrations of PICP and ICTP in serum during treatment in responders. The area between the dotted lines indicates the reference interval.

**Figure 2** The concentrations of PICP and ICTP (log-scale) in serum during treatment in patients with an initial remission and a later recurrence. The filled symbols present the values at the time of renal failure. The area between the dotted lines indicates the reference interval.

*Patients with recurrence*

Four patients first responded to the therapy with a decrease in the concentration of M protein. Before treatment, the urinary Ca/Cr was elevated in two of these patients, urinary OHP/Cr and serum AP only in one patient. The pretreatment level of ICTP was elevated in every patients, whereas the PICP concentration was within the reference range (Table I, Figure 2). At the time of recurrence (at 12, 18, 24 and 30 months, respectively), the bone lesions were found to progress, and the M protein and ICTP concentrations increased. PICP was above the upper limit of the reference interval in only one patient. Three patients died, having hypercalcaemia in the terminal phase. In one of them renal function was impaired (creatinine 182 $\mu mol\ l^{-1}$, calcium 3.93 $mmol\ l^{-1}$ and ICTP 17.9 $\mu g\ l^{-1}$) but could be restored again by hydration and calcitonin infusions (creatinine 119 $\mu mol\ l^{-1}$, calcium 2.14 $mmol\ l^{-1}$ and ICTP 90.5 $\mu g\ l^{-1}$).

*Nonresponders*

In all four patients bone disease progressed leading to death. Two patients developed hypercalcaemia; renal function worsened in both of them. The fasting urinary Ca/Cr ratio was increased at the beginning of treatment in all these patients. The fasting OHP/Cr ratio was elevated in three patients and the serum AP activity was normal (Table I). The serum PICP concentrations were within the normal range, whereas the serum ICTP levels were high (Table I, Figure 3). The highest ICTP values were seen in the patients with hypercalcaemia and renal failure (ICTP 43.3 $\mu g\ l^{-1}$, calcium 2.87 $mmol\ l^{-1}$, creatinine 159 $\mu mol\ l^{-1}$ and ICTP 33.5 $\mu g\ l^{-1}$, calcium 2.69 $mmol\ l^{-1}$, creatinine 661 $\mu mol\ l^{-1}$, respectively).

*Correlations and regressions*

No positive correlation was found between the concentrations of ICTP and PICP either before or during the treatment. When the pretreatment levels of different markers, including that of the M protein, were correlated with the number of bone lesions, there was a positive relationship between bone lesions and ICTP ($r = 0.58$; $P = 0.02$) and between bone lesions and OHP/Cr ($r = 0.55$; $P = 0.03$). When the method of all possible subset regression was used to explain the number of bone lesions by the markers, the most significant marker seemed to be ICTP ($C_p = 4.1$; $R_2 =$ 34%). The best two marker subset ($C_p = 1.5$; $F = 7.06$, $P =$ 0.0094) was ICTP ($t = 2.9$; $P = 0.01$) and M-protein ($t = 2.3$; $P = 0.04$). This model explained 54% of the variance of the lesions. Of all the markers, ICTP was the only statistically significant predictor for survival (coeff/SE = 2.1; $P < 0.05$).

### Discussion

Remodelling of bone first comprises a phase of osteoclast activation and osteoclastic bone resorption with the formation of a resorption cavity. Subsequently, osteoblasts synthesis type I collagen. Then the osteoid matrix thus formed undergoes mineralisation and self-repair of skeletal tissue occurs. In myeloma, the amount of bone deposited in a resorption cavity does not equal that removed (Kanis *et al.*, 1988). As a result, excessive bone resorption occurs, leading to a disproportionally increased concentration of ICTP, which is a cross-linked peptide, liberated into the circulation

**Figure 3** The concentrations of PICP and ICTP in serum during treatment in nonresponders. The filled symbols present the values at the time of renal failure. The area between the dotted lines indicates the reference interval.

during type I collagen degradation. In this study, the ICTP concentration correlated with the number of osteolytic lesions as well as did the OHP/Cr ratio. ICTP was also superior to M protein, when these markers were correlated with survival. It is known that production of the M component predicts the stage of the disease with resonable accuracy, but as a single parameter it does not predict survival (Durie & Salmon, 1975). This is explained by the fact that a high M component level can be due to either a small number of cells producing large quantities of the M component per cell or a large number of cells producing only a small amount of the M component per cell.

In our patients ICTP behaved like a tumour marker: elevated levels indicated a progressive or recurring disease, whereas decreasing levels were associated with a regressive disease. The ICTP concentration observed before treatment was high in the patients who later turned out to be nonresponders or who got a relapse after an initial remission. Thus, an initially high ICTP value seems to predict the prognosis in the patients who fail to respond to the treatment. Finding an elevated ICTP concentration could thus allow clinicians to use more aggressive chemotherapy or to combine other drugs to the treatment, such as bisphosphonates, which inhibit osteoclastic bone resorption (Elomaa *et al.*, 1983; Merlini *et al.*, 1990). Since the number of patients in the present study is small, we will test the validity of this conclusion in a much larger group of patients, participating in a randomised study on the effect of clodronate, combined with the ordinary treatment with melphalan and prednisolon.

Before interpreting changes in serum ICTP concentration, one should remember that ICTP is cleared by kidney and renal failure may influence the values. Nevertheless, after restoration of renal function we noted the highest ICTP value which increased simultaneously with progressive osteolysis. We have not seen high ICTP levels without excessive bone resorption. On the other hand, we have no experience of ICTP values in patients who have acute renal failure without bone disease. Using the methods of calcium kinetics and dynamic histomorphometry, we have shown a significant correlation between serum ICTP concentration and bone resorption in patients with low and high bone turnover rates (Eriksen *et al.*, 1992).

The lack of correlation between ICTP and PICP indicates unbalanced bone turnover and suggests unchanged or even depressed bone formation in multiple myeloma. This is in accordance with clinical experience, since in myeloma the osteolytic holes rarely heal during chemotherapy. Corticosteroids are known to inhibit osteoblast activity and collagen synthesis (Lykert & Raisz, 1990; Canalis, 1983; Nielsen *et al.*, 1988). Because the samples here were taken before each treatment course, the low or normal levels of serum PICP in myeloma patients indicate an inhibition of osteoblast function by the disease rather than the effect of corticosteroids. Indeed, it has been recently shown that the concentration of osteocalcin in serum correlates inversely with the severity of multiple myeloma, the lowest values being observed in patients with extensive lytic lesions with frequent hypercalcaemia (Bataille *et al.*, 1990).

Urinary hydroxyproline excretion is strongly affected by dietary collagen; in principle this is also possible for the urinary pyridinoline cross-links, although they do not appear to be absorbed after gelatin load (Colwell *et al.*, 1990). PICP, on the other hand, is a large (Mr 100,000) globular protein released from type I procollagen during the extracellular phase of collagen biosynthesis. The serum ICTP antigen is a composite of three cross-linked peptides, have a Mr of more than 9,000, and released by proteolytic digestion of type I collagen fibres during bone resorption. PICP and ICTP are thus too large to be absorbed in antigenically intact form from the diet.

Another advantage of ICTP as a marker of bone resorption over hydroxyproline or the pyridinoline cross-links is the fact that ICTP still carries information about the collagen type it originates from. Hydroxyproline is present in all collagenous proteins and in some other proteins, e.g. the C1q component of complement. The pyridinoline cross-links are more selective, as they are formed during the maturation of collagen fibres in bone and cartilage, e.g. in the collagen types I and II. Although type I collagen is also found in large quantities in soft tissues, the structure of its mature cross-links e.g. in skin differs from that in bone (Mechanic *et al.*, 1987). Thus it is likely that skin collagen degradation does not lead to liberation of antigens reacting in the ICTP assay. However, this question warrants further study.

In conclusion, the concentration of the cross-linked carboxyterminal telopeptide of type I collagen (ICTP) is a sensitive and specific bone resorption marker, which strongly correlates with the number of bone lesions and the survival in multiple myeloma. In contrast to ICTP, the concentration of PICP, which is derived from type I collagen synthesis, is relatively low, suggesting unchanged or even depressed bone formation. Simultaneous use of these serum markers of type I collagen metabolism offers an additional and easy means to follow bone turnover and evaluate the response to therapy in multiple myeloma.

### References

BATAILLE, B., DELMAS, P., CHAPPARD, D. & SANY, J. (1990). Abnormal serum bone GLA protein levels in multiple myeloma. Crucial role of bone formation and prognostic implications. *Cancer*, **66**, 167–172.

CANALIS, E. (1983). Effect of glucocorticoids on type I collagen synthesis, alkaline phosphatase activity, and desoxyribonucleic acid content in the rat calvariae. *Endocrinology*, **112**, 931–939.

COLWELL, A., EASTELL, R., ASSIRI, A.M.A. & RUSSELL, R.G.G. (1990). Effect of diet on deoxypyridinoline excretion. In *Osteoporosis*. Christiansen, C. & Overgaard, K. (eds), Osteopress ApS: Copenhagen, pp. 590–591.

DRAPOR, N.R. & SMITH, H. (1981). *Applied Regression Analysis*, 2nd edition, Wiley: New York, pp. 294–332.

DURIE, B.M.G. & SALMON, S.E. (1975). A clinical staging system for multiple myeloma. *Cancer*, **36**, 842–854.

ELOMAA, I., BLOMQVIST, C., GRÖHN, P., PORKKA, L., KAIRENTO, A.-L., SELANDER, K., LAMBERG-ALLARDT, C. & HOLMSTRÖM, T. (1983). Long-term controlled trial with diphosphonate in patients with osteolytic bone metastases. *Lancet*, **i**, 146–149.

ERIKSEN, E.F., CHARLES, P., MOSEKILDE, L., RISTELI, L. & RISTELI, J. (1992). Serum markers of type I collagen formation and degradation in metabolic bone disease: correlation to bone histomorphometry. *J. Bon. Min. Res.* (in press).

GARRETT, R., DURIE, B.G.M., NEDWIN, G., GILLESPIE, A., BRINGMAN, T., SABATINI, M., BERTOLINI, D. & MUNDY, G. (1987). Production of lymphotoxin, a bone resorbing cytokine by cultured human myeloma cells. *N. Engl. J. Med.*, **317**, 526–532.

KANIS, J.A., MCCLOSKEY, E.W., THARAVAJAH, M., EVANS, D., HAMDY, N.A.T., PRESTON, E. & GRAEVES, M. (1988). Calcium metabolism and myeloma, and the treatment of hypercalcemia. *Hematol. Oncol.*, **6**, 77–81.

KRAENZLIN, M.E., TAYLOR, A.K. & BAYLINK, D.J. (1989). Biochemical markers for bone formation and bone resportion. In *Clinical Impact of Bone and Connective Tissue Markers*, Lindh, E. & Thorell, J.I. (eds), Academic Press: London. pp. 289–303.

LYKERT, B.P. & RAISZ, L.G. (1990). Glucocorticoid-induced osteoporosis: pathogenesis and management. *Ann. Int. Med.*, **112**, 352–364.

MECHANIC, G.L., KATZ, E.P., HENMI, M., NOYES, C. & YAMAUCHI, M. (1987). Locus of a histidine-based, stable trifunctional, helix to helix collagen cross-link: stereospecific collagen structure of type I skin fibrils. *Biochemistry*, **26**, 3500–3509.

MELKKO, J., NIEMI, S., RISTELI, L. & RISTELI, J. (1990). Radioimmunoassay of carboxyterminal propeptide of human type I procollagen. *Clin. Chem.*, **36**, 1328–1332.

MERLINI, G., PARRINELLO, G.A., PICCININI, L., CREMA, F., FIORENTINI, M.L., RICCARDI, A., PAVESI, F., NOVAZZI, F., SILINGARDI, V. & ASCARI, E. (1990). Long-term effects of parenteral dichloromethylene bisphosphonate ($Cl_2$ MBP) on bone disease of myeloma patients treated with chemotherapy. *Haematol. Oncol.*, **8**, 23–30.

NIELSEN, H.K., CHARLES, P. & MOSESKILDE, L. (1988). The effect of single oral doses of prednisone on the cicardian rhytm of serum osteocalcin in normal subjects. *J. Clin. Endocrinol. Metab.*, **67**, 1025–1030.

RISTELI, J., NIEMI, S., ELOMAA, I. & RISTELI, L. (1991). Bone resorption assay based on a peptide liberated during type I collagen degradation. *J. Bone Min. Res.*, **6** (suppl), S251.

SIMON, L.S., KRANE, S.M., WORMAN, P.D., KRANE, I.M. & KOVITS, K.L. (1984). Serum levels of type I and III procollagen fragments in Paget's disease of bone. *J. Clin. Endocrinol. Metab.*, **58**, 110–120.